# Impact of Partner-Related Social Harms on Women's Adherence to the Dapivirine Vaginal Ring During a Phase III Trial

**DOI:** 10.1097/QAI.0000000000001866

**Published:** 2018-09-18

**Authors:** Thesla Palanee-Phillips, Sarah T. Roberts, Krishnaveni Reddy, Vaneshree Govender, Logashvari Naidoo, Samantha Siva, Zakir Gafoor, Arendevi Pather, Flavia Matovu, Kudzai Hlahla, Bonus Makanani, Gonasagrie Nair, Katie Schwartz, Kristine Torjesen, Elizabeth Brown, Lydia Soto-Torres, Jared Baeten, Elizabeth T. Montgomery

**Affiliations:** * Wits Reproductive Health and HIV Institute (Wits RHI), Johannesburg, South Africa;; † Women's Global Health Imperative (WGHI), RTI International, San Francisco, CA;; ‡ South African Medical Research Council, Durban, South Africa;; § Johns Hopkins University Research Collaboration, Makerere University, Kampala, Uganda;; ║ Clinical Trials Unit, College of Health Sciences, UZCHS-CTU University of Zimbabwe, Harare, Zimbabwe;; ¶ Johns Hopkins University Research Project, Malawi College of Medicine, Queen Elizabeth Central Hospital, Blantyre, Malawi;; # Center for AIDS Programme of Research in South Africa (CAPRISA); Durban, South Africa;; ** FHI 360, Durham, NC;; †† Statistical Center for HIV/AIDS Research and Prevention, Fred Hutchinson Cancer Research Center, Seattle, WA;; ‡‡ National Institute of Allergy and Infectious Diseases, Washington, DC; and; §§ Departments of Global Health, Medicine, and Epidemiology, University of Washington, Seattle, WA.

**Keywords:** social harms, HIV prevention, pre-exposure prophylaxis, adherence, intimate partner violence

## Abstract

**Background:**

Long-acting female-initiated methods such as the dapivirine ring may give women greater agency in HIV-1 prevention. However, social harms, defined as nonmedical adverse consequences of study participation or dapivirine ring use, may reduce product adherence and consequently HIV-1 protection.

**Methods:**

We assessed whether experiencing social harms from male partners was associated with lower adherence to the dapivirine ring in the MTN-020/ASPIRE trial. Reports of social harms were solicited quarterly. Low adherence was defined by plasma dapivirine levels ≤95 pg/mL or residual dapivirine levels in returned rings >23.5 mg.

**Results:**

Among 2629 women enrolled in ASPIRE, 85 (3.2%) reported 87 social harms during a median follow-up of 1.6 years. Women were significantly more likely to have low adherence, measured by plasma dapivirine levels, at visits with a social harm in the past month than at visits where no social harm was reported (adjusted risk ratio 2.53, 95% confidence interval: 1.37 to 4.66, *P* = 0.003). There was no association for social harms reported ≥1 month prior, suggesting an acute, short-term effect. Women were significantly more likely to not return a ring at visits with a social harm reported (adjusted risk ratio 24.70, 95% confidence interval: 18.57 to 32.85, *P* < 0.001). In rings that were returned, social harms were not associated with residual dapivirine levels.

**Conclusions:**

Although social harms were uncommon (<5% of women with >1 year of use), participants reporting social harms by male partners had lower adherence to the dapivirine ring. Strategies to mitigate nonadherence to product use related to social harms should be evaluated in future studies of female-controlled HIV-1 prevention options.

## INTRODUCTION

The MTN-020/ASPIRE trial and the IPM 027/The Ring Study showed that the dapivirine vaginal ring, a monthly female-initiated HIV-1 prevention method, provides some protection against HIV-1 acquisition.^1,2^ However, like for other biomedical prevention strategies, consistent adherence is needed for the ring to effectively reduce risk of HIV-1 infection.^3^

To maximize the potential public health impact of the dapivirine vaginal ring, an important priority is to identify and address factors that impede women's uptake and consistent use. One set of well-recognized factors includes male partner influence and social harms (SHs). Although designed to enable greater female autonomy, many women's ability and willingness to use topical microbicides or pre-exposure prophylaxis (PrEP) is influenced by their male sexual partners.^4–7^ Although male partner support can improve adherence, negative aspects such as relationship discord and intimate partner violence (IPV) have been associated with low adherence to both antiretroviral therapy for treatment and oral PrEP for prevention.^7–11^ Although rates of SHs are generally low in HIV prevention trials, they are recognized as important barriers to trial participation and future uptake of products under investigation.^12–16^ It is unclear whether experience of SHs may also inhibit adherence to microbicide products, thereby reducing women's autonomy to protect themselves against HIV-1 infection.

We assessed the frequency, recurrence, and severity of reports of SHs in the ASPIRE trial and evaluated how women reporting these experiences differed from other participants in regards to key characteristics. We also examined the association of SHs with low adherence and other indicators of suboptimal ring use. These results provide insight into how measurement and monitoring of SHs in future roll-out activities of the ring may identify women requiring additional psychosocial support to mitigate adherence challenges.

## METHODS

### Study Population

The ASPIRE trial design, population, procedures, and primary findings have previously been described.^1,17^ ASPIRE was a phase III, randomized, placebo-controlled trial that evaluated the safety and effectiveness of a dapivirine vaginal ring for the prevention of HIV-1 infection in African women.^17^ ASPIRE was conducted between August 2012 and June 2015 and enrolled 2629 HIV-uninfected women at 15 sites in Malawi, South Africa, Uganda, and Zimbabwe. Women were randomly assigned in a 1:1 ratio to receive either a silicone elastomer vaginal matrix ring containing 25 mg of dapivirine or a placebo vaginal ring, and followed monthly for a minimum of 1 year (median, 1.6 years; interquartile range, 1.1–2.3 years). All women received a package of free HIV prevention services, including risk-reduction counseling, male and female condoms, partner HIV testing and referrals, and treatment of sexually transmitted infections for participants and partners. The trial protocol was approved by all participating sites' institutional review boards.^1^ All participants provided written informed consent in English or their local language.

### Data Collection

SHs were defined as nonmedical adverse consequences of dapivirine vaginal ring use or of trial participation more generally. All SH assessments were conducted one-on-one with a trained interviewer in a private setting. At quarterly trial visits, participants were asked a standardized question in face-to-face interviews: “At any time during the past 3 months, have you experienced a SH related to your study participation?” Women could also spontaneously report SHs at any visit. The assessment procedures were consistent across visits and study sites. When a SH was reported, the event was documented on a structured case report form called the “Social Impact Log.” The log included open-ended questions for a description of the event and the onset date, followed by close-ended questions on whether the event involved physical harm to her or her children, and the impact on her quality of life (minimal disturbance, moderate disturbance with no significant impact, or major disturbance with significant impact). These 3 categories were discussed with the participants when assessing impact on her life. Staff also characterized the SH as being related to family, sex partner, other personal relationships, travel/immigration, employment, education, medical/dental, housing, or other. Characterization of SHs was generally based on the participant's own perception. Uncertainties related to definition of SHs were managed between the site leadership, the study management team, and the protocol safety review team, which comprised the study leadership and safety clinicians.

To objectively assess product adherence, plasma samples were collected at quarterly visits and tested for the presence of dapivirine using a validated ultra-performance liquid chromatography–tandem mass spectrometry assay (Clinical Pharmacology Analytical Laboratory), with a lower limit of quantification of 20 pg/mL. After the first year of the trial, testing for residual dapivirine in used rings (returned to the clinic each month) was initiated with the use of acetone extraction and high-pressure liquid chromatography (Parexel).^1^ At each monthly visit, study staff also documented whether the ring was returned to the study clinic, whether it was in place within the vagina at the start of the visit, and whether the participant accepted or declined a new ring at that visit.

Data collected in face-to-face interviews at trial enrollment included demographic characteristics (*age*, *education*, *employment*, *income*, *alcohol intake*, *and marital status*), sexual behavior (*partnership status*, *coital frequency*, *condom use*, *and outside partnerships*), characteristics of the primary sex partner (*HIV status*, *participant belief that he has other partners*, *and visits to the study clinic*), disclosure of study participation and ring use to the primary sex partner, and worries about ring use, including worries that her primary sex partner might feel the ring or not approve of the ring. Sensitive questions about alcohol, condom use, other sexual partners, and worries about the ring were also asked in audio computer-assisted self-interview (ACASI) questionnaires to minimize underreporting.

During follow-up, testing for HIV-1 and pregnancy was performed monthly, as previously described.^1^ Data on sexual behavior, disclosure of study participation and ring use, and primary sex partner characteristics were collected quarterly through interviewer-administered questionnaires.

### Data Analysis

Analyses were restricted to SHs that were partner-related because these accounted for the vast majority of all SHs reported. The Kaplan–Meier method was used to estimate the cumulative incidence of the SH during the study. We explored baseline predictors of experiencing any SH during the study using bivariate Poisson regression models with robust standard errors to estimate risk ratios (RRs). To identify the strongest predictors, all the variables that were significantly associated with SHs at *P* < 0.05 in bivariate analyses were included in a multivariable model. This model also controlled for country a priori as a potentially important confounder.

In the adherence analysis, women were categorized at each visit as having no or any SH reported to date in the study. For women who reported SHs, visits were further categorized by whether the most recent harm had occurred <1 month ago, 1–3 months ago, or >3 months ago.

Our primary measure of low adherence was defined as plasma dapivirine ≤ 95 pg/mL at that quarterly visit, a level consistently achieved within 8 hours of continuous use. This threshold was chosen to aid in distinguishing cases in which the ring was removed during the month and then reinserted the morning of a clinic visit. In addition, we characterized high adherence as <23.5 mg dapivirine in the returned ring (ie, with >1.5 mg released) in the subset of monthly visits with returned vaginal rings. Because both measures were based on dapivirine levels, they are only valid for participants who received the active (dapivirine) ring and not for those in the placebo arm.

We evaluated the associations between the SH and each ring adherence measure using bivariate and multivariable (adjusted) generalized estimating equation Poisson models with an exchangeable correlation matrix and robust standard errors, to account for repeated measures for each participant.^18,19^ All models excluded participants in the placebo arm and visits with study-initiated product holds (ie, for seroconversion, pregnancy or breastfeeding, or safety concerns). Multivariable models adjusted a priori for age, study site, and time in the study. We also evaluated the following covariates as potential confounders and retained them in the model if they resulted in meaningful changes (>10%) to the estimated RRs: baseline covariates of employment status, alcohol use, belief that her primary partner has additional sex partners, partner-related worries about ring use and time varying covariates of partnership status, disclosure of ring use to primary partner, knowledge of primary partner's HIV status, and whether the participant had >1 sexual partners in the past month. Because the amount of missing data for the exposure and covariates was small (<5% of visits), we conducted complete case analyses.

One potential consequence of the SH is relationship interruption or dissolution, with potential reduction in sexual activity. Lack of sexual activity has been associated with low adherence to prophylaxis interventions in other studies.^20,21^ To understand whether sexual activity mediated the relationship between the SH and ring adherence, we repeated our analysis of the SH and plasma dapivirine levels restricted to participants who had reported any sexual activity in the 7 days before the plasma sample was collected.

Additional analyses examined the association between the SH and whether the ring was in place at the start of the study visit, whether the used ring was returned to the clinic, and whether the participant declined a new ring at the study visit. These analyses were conducted using the same methods as the biomarker adherence analyses described above, except that data were available for all monthly follow-up visits and from study participants in the placebo and active arms. As above, we excluded visits with study-initiated product holds.

## RESULTS

### Baseline Participant Characteristics

Two thousand six hundred twenty-nine women were enrolled in the ASPIRE trial. The median age was 26 years (interquartile range 22–31) and the majority (59%) were unmarried.^17^ At baseline, nearly 100% of participants reported having a primary sex partner in the prior 3 months, but 43% did not know his HIV-1 status; 17% reported additional concurrent partners. Nearly two-thirds (64%) reported having disclosed to primary partners about planned vaginal ring use in the trial.^17^

### SHs Occurring During the Study

During ASPIRE, 94 SHs as a result of dapivirine vaginal ring use or trial participation were reported over 4680 person years of follow-up, of which 87 (92.6%) were partner-related (Table 1). Eighty-five women (3.2% of total) reported a partner-related SH over an average follow-up of >1.5 years. Two women reported 2 partner-related SHs and the remainder (n = 83) reported 1 each. Of these 85 women, 52 (61%) had disclosed study participation to their primary partner at enrollment.

**TABLE 1.**
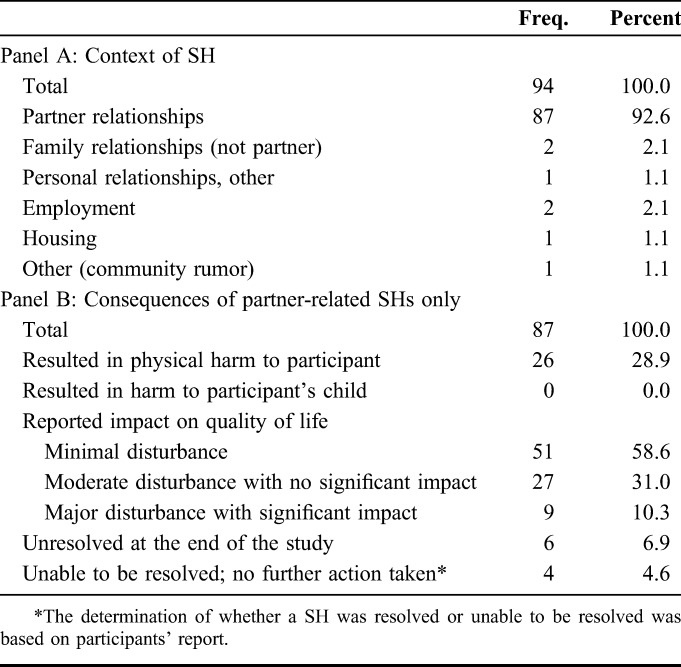
Summary of SHs Reported in ASPIRE

Twenty-six (28.9%) resulted in physical harm to the participant, and there were no reports of harm to participant's children. Most partner-related SHs were reported to have minimal impact on quality of life (n = 51, 58.6%), with 27 (31.0%) classified as moderate disturbance and 9 (10.4%) causing a major disturbance with significant impact. Common triggers of SHs included discovery of the ring during sex or foreplay (n = 23, 26.4%), partner notification of a sexually transmitted infection (n = 4, 4.6%), and partner suspicion that the ring was associated with witchcraft, promiscuity, or ill health (n = 16, 18.4%). The most common consequences were removal or destruction of the ring by the partner, physical and verbal violence, and/or relationship dissolution. One SH was reported after seroconversion to HIV-1 (and was related to her HIV-1 status) and none were reported during pregnancy.

### Correlates of SH

Table 2 shows the proportion of women in the study who experienced the SH by demographic and behavioral characteristics using bivariate and multivariable analyses. When controlling for other factors, the only demographic characteristic predictive of the SH was age; the probability of experiencing a SH during the study decreased with increasing age (*P* ≤ 0.001) with women aged 18–26 years more than 3 times more likely to experience a SH. Although many demographic (eg, being unmarried and not earning her own income), partner-related (eg, primary partner was not aware of their study participation or ring use at enrollment), risk-related (eg, women with a new primary sex partner in the past 3 months), and attitudinal (eg, being worried that her partner would feel the ring or not like the ring) factors were associated with SHs in bivariate analyses, only younger age remained statistically significant in the multivariable model.

**TABLE 2.**
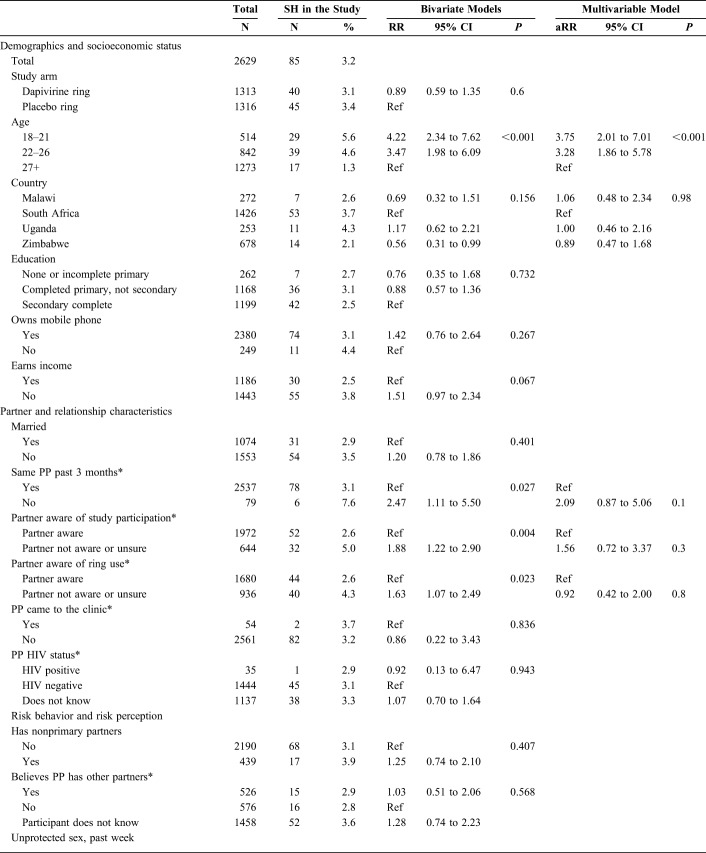

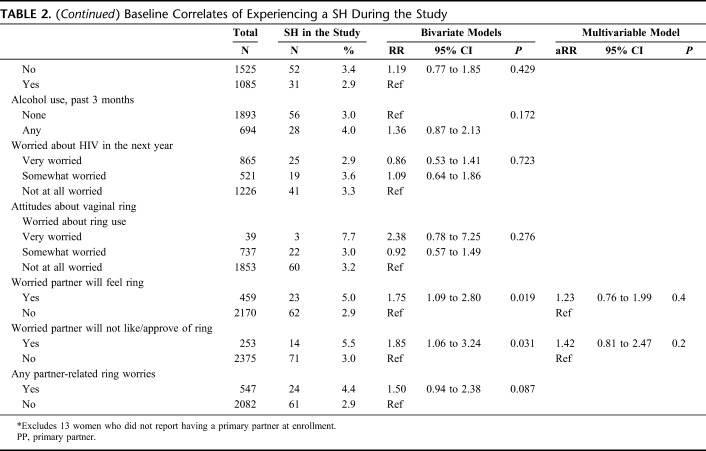
Baseline Correlates of Experiencing a SH During the Study

### Association Between SH and Ring Adherence

Among the 1313 women in the active arm of the study, there were 9057 visits with a plasma dapivirine measure. Low adherence, defined by plasma dapivirine ≤95 pg/mL, was detected at 15.4% of visits among participants who had not reported a partner-related SH to date in the study (Table 3). In comparison, low adherence was detected at 27.8% of visits with any SH before that visit, including 52.9% of visits with a SH reported in the past month, 19.2% of visits with a SH reported 1–3 months ago, and 26.2% of visits with a SH reported >3 months ago (Table 3). Adjusting for age, study site, and time on the study, women were 2.53 times more likely to have low adherence at visits when a SH was reported in the past month, compared with visits where no SH was reported to date [95% confidence interval (CI): 1.37 to 4.66, *P* = 0.003]. RRs for the SH 1–3 months ago or >3 months ago were not significantly different from 1.0, suggesting that the effect was limited to the first month following the reported SH.

**TABLE 3.**
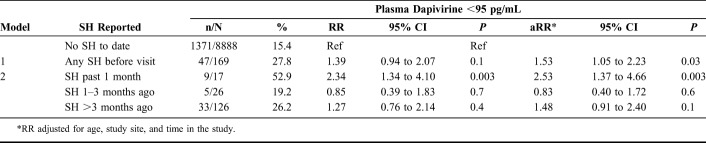
Association Between SHs and Adherence by Plasma Dapivirine Levels

Seventy-five percent of participants reported sexual activity in the 7 days before the adherence measurement. Among this subgroup, the association between a SH in the past month and low adherence was similar to the overall sample [adjusted RR (aRR) 2.63, 95% CI: 1.29 to 5.34, *P* = 0.008], suggesting that low adherence was not driven by changes in sexual activity following the SH occurrence.

Ring concentration data were missing at 1.9% of monthly visits (414/21,707) for active-arm participants after ring testing was initiated. Residual ring concentration data were missing for a significantly higher proportion of visits among women who had a SH in the past month (15/33 or 45.5%, *P* < 0.001) or >1 month ago (14/381 or 3.7%, *P* < 0.02) than for visits among women with no SH to date (385/21,293 or 1.8%). The most common reason for missing ring concentration data was that the ring was not returned to the clinic. This explained 100% of missingness for women with SHs in the past month, 57% of missingness for women with SHs >1 month ago, and 40% of missingness overall.

Among the rings that were tested, residual dapivirine levels in rings that were tested indicated low to no adherence (>23.5 mg) at 15.4% of visits among women with no SH to date and 25.7% of visits among women who ever reported a SH (Table 4, Panel A). There were no statistically significant associations between SHs and residual dapivirine levels in rings in any timeframe (Table 4, Panel B). Under the assumption that residual ring data would have indicated low adherence at all visits with unreturned rings, had they been returned and tested, the aRRs for low adherence among women were very similar to the estimates from the model based on plasma dapivirine levels: 2.47 (95% CI: 1.62 to 3.77, *P* < 0.001) for women with a SH in the past 1 month, and not significantly different from 1.0 for women reporting SHs 1–3 months ago or >3 months ago (Table 4, Panel B).

**TABLE 4.**
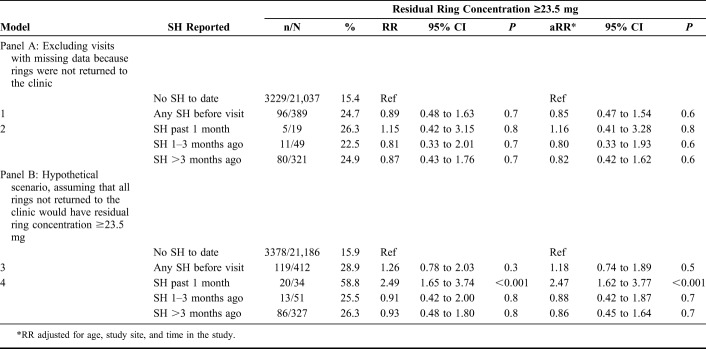
Association Between SHs and Adherence by Residual Ring Concentration

As can be inferred from the above data, there was a strong association between a SH in the past month and whether the ring was returned to the clinic or in place at the start of a subsequent visit (Table 5). Among women reporting no SH, the ring was not observed to be in place at the start of the visit at 2.4% of visits, and no used rings were returned to the clinic at 1.2% of visits. However, women who had experienced a SH in the past month were 15 times more likely to not have the ring in place at the start of the visit (46.1% of visits, aRR 14.91, 95% CI: 11.50 to 19.33, *P* < 0.001) and 25 times more likely to not return their used rings to the study clinic (36.5% of visits, aRR 24.70, 95% CI: 18.57 to 32.85, *P* < 0.001). SHs that occurred 1–3 months before the visit were also significantly associated with these outcomes, although with substantially smaller RRs (aRR 2.71 for ring not in place, 95% CI: 1.57 to 4.71, *P* < 0.001; aRR 2.39 for ring not returned, 95% CI: 1.00 to 5.67, *P* = 0.049).

**TABLE 5.**
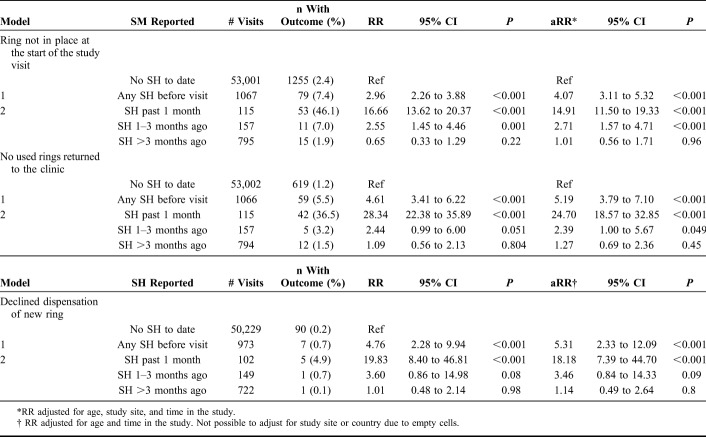
Association Between SHs and Secondary Measures of Ring Adherence

It was rare for participants to decline dispensation of a new ring in ASPIRE: Of 51,614 scheduled visits at which a ring could have been dispensed, rings were declined at only 105 visits (0.2%) (Table 5). Nevertheless, women were more likely to decline the ring if they had experienced a SH, particularly in the first month after the SH (aRR 18.18, 95% CI: 7.39 to 44.70, *P* < 0.001). There was also indication of elevated risk of ring declines in the 1–3 months after a SH, but the result was not statistically significant (aRR 3.46, 95% CI: 0.84 to 14.33, *P* = 0.09).

## DISCUSSION

Many HIV prevention trials are conducted in areas where women are marginalized in terms of decision-making around sexual and reproductive health. SHs are commonly associated with both poor health and HIV outcomes.^8–10^ In the context of biomedical HIV prevention interventions, SHs may impose limitations on use and consequently impact adherence. Although reported SHs were relatively uncommon in this study (<5%), most were partner-related (93%). Women reporting partner-related SHs in the past month were significantly more likely to have low adherence to ring use, measured by plasma dapivirine levels, compared to women with no SHs.

This is the first study to examine the association between partner-related SHs and vaginal ring use adherence. By definition, reported SHs were related to study participation, and thus non–study-related experiences of gender-based violence and IPV were not measured. Nevertheless, our findings are consistent with a study among African HIV serodiscordant couples showing that IPV increases the risk of low adherence to oral PrEP,^7^ and with other studies that have highlighted the role of violence and relationship discord as a barrier to disclosure and product use.^4,22,23^ Although these studies suggest that women should or would be more likely to use HIV prevention products covertly to reduce the risks associated with disclosure and partner disapproval, a complementary body of work highlights the importance of partner support and disclosure of product use in promoting adherence to PrEP and microbicides^4,24–26^ and emphasizes that many women regard their partner's knowledge and involvement in the use of female-initiated HIV prevention as desirable, culturally appropriate, or necessary to preserve relationship harmony.^4^ Qualitative findings from an oral PrEP trial that enrolled heterosexual serodiscordant couples indicated that adherence is improved when partners, irrespective of gender, provide support.^24^ Ultimately, whether male partners positively or negatively influence ring adherence will be dependent on relationship dynamics and both partners' characteristics. In the context of these unique circumstances, counselors should aim to support women's own decisions on how to use HIV prevention methods safely and consistently.

We found that women aged 18–21 years had a significantly higher likelihood of reporting SHs during the study than older women. In post hoc analyses of the ASPIRE trial, the ring did not provide effective protection against HIV-1 for women in this younger age group (−27% efficacy; 95% CI: −133 to 31; *P* = 0.45), but had 56% efficacy among women >21 years (95% CI: 31 to 71; *P* < 0.001), a difference that was correlated with adherence levels measured by dapivirine in returned rings or plasma, as previously described.^1^ As recent research and programmatic data have highlighted, young women also experience disproportionately high rates of HIV infection, in comparison with their male counterparts.^27,28^ This is due in part to biological factors, but also to the relative lack of power that younger women have in relationships, particularly if they are age disparate^27–31^ or with perpetrators of IPV.^32,33^ Epidemiologic studies suggest that women's risk of HIV is higher in the context of IPV due to both diminished control of sexual protection among abused women and increased likelihood of HIV infection among men who perpetrate IPV.^33,34^ Sociocontextual issues such as exposure to a partner-related SH may have contributed to lower adherence and a lack of HIV-1 protection in women between 18 and 21 years in ASPIRE, while concurrently contributing to increased risk of HIV-1 infection.

We did not detect an association between the SH and ring adherence as measured by residual dapivirine levels in rings. However, there is a high likelihood of bias in this analysis because residual ring data were only available for 55% of visits occurring in the month after a SH was reported, compared with 98% of visits overall. This may be due to the fact that women with a recent SH were 15–25 times more likely to return to the clinic without the ring at all, or without it in place. Narrative accounts on SH case report forms as well as qualitative data from ASPIRE described male partners removing and destroying or discarding rings.^35^ Visits with low adherence resulting from these actions had to be omitted from the residual ring analysis because the rings were not available to be tested and may explain our null finding. This conclusion is supported by the hypothetical scenario that if all unreturned rings did have test results suggesting low adherence, the findings from the residual ring analysis would have supported those from the plasma dapivirine analysis.

In future ring research and programmatic activities—whether used for an HIV-1 prevention indication or potentially even in a multipurpose prevention context—the nonreturn or decline of rings might be used by clinicians or counselors as a potential signal to probe about experiences of SHs and IPV. In the HIV Open label Prevention Extension (HOPE) study, the open-label extension of ASPIRE, trial participants are supported to not accept the ring, should they so choose but are still permitted access to study-related services. Further analyses of differences between individuals accepting and declining the ring, including partnership dynamics and experiences of SHs/IPV, will offer another opportunity to examine this association.

We found that when more than 1 month had passed after the SH, adherence seemed to return to presocial harm levels. These improvements could have been due to counseling and other interventions by study staff, shifting behaviors or attitudes as a response to the SH (eg, changes in risk perception), or greater awareness of risk and increased efforts related to adherence. The SH may have also occurred either as a result of initial disclosure or triggered a disclosure (eg, if covert ring use was discovered), which may have made subsequent ring use easier.

Study strengths include a large sample size and a prospective study design. An important limitation of the study is that classification of a SH was based on self-report and may have been underreported. Women may not have disclosed a SH if they did not consider specific acts to be abusive, did not feel comfortable discussing SHs with the study staff, or feared being exited from the trial. Furthermore, in this study, the SH captured participant experiences of gender-based violence, including IPV, if (and only if) the participant or study staff determined that the violence was associated with study participation. Gender-based violence and IPV specifically are highly prevalent in the ASPIRE research settings,^1,29,33,36–39^ and the report of SHs thus represents only a subset of violence or relationship discord in these participants' lives. If the degree of underreporting of a SH was the same among women with low versus high ring adherence, this would likely underestimate the risk of low adherence associated with the SH. A second limitation was that the residual drug assessments were only initiated a year into the study and are an incomplete data set. However, there was no evidence that the prevalence of a SH was different before and after ring testing was initiated. Finally, although biomarkers of adherence are likely to be more accurate than self-report or clinic-based product returns, they remain imperfect measures. Both the plasma and residual ring dapivirine levels may overestimate adherence because participants may be characterized as adherent to ring use, although they may have used the ring for only a portion of the month. Interindividual variability in the rate of dapivirine release from the ring might confound objective assessments of adherence.

## CONCLUSIONS

SHs from male partners can represent an important barrier to HIV prevention product use. Insofar as HIV risk is correlated with IPV, and both SHs and IPV are associated with reduced adherence, HIV prevention interventions that engage women, men, and couples with concomitant behavioral (eg, counseling and support services) and biomedical (eg, ring and oral PrEP) components may be efficient public health investments.
